# Elucidation of Geniposide and Crocin Accumulation and Their Biosysnthsis-Related Key Enzymes during *Gardenia jasminoides* Fruit Growth

**DOI:** 10.3390/plants12112209

**Published:** 2023-06-03

**Authors:** Luhong Zhang, Yang Ai, Yunzhu Chen, Changzhu Li, Peiwang Li, Jingzhen Chen, Lijuan Jiang, Yuhong Pan, An Sun, Yan Yang, Qiang Liu

**Affiliations:** 1 College of Life Science and Technology, Central South University of Forestry and Technology, Changsha 410004, China; 20210100019@csuft.edu.cn (L.Z.); aiyang9968@163.com (Y.A.); znljiang2542@163.com (L.J.); 20221100112@csuft.edu.cn (Y.P.); m15111406060@163.com (A.S.); 2 State Key Laboratory of Utilization of Woody Oil Resource, Hunan Academy of Forestry, Changsha 410004, China; cyzcarol@foxmail.com (Y.C.); lichangzhu2013@aliyun.com (C.L.); lindan523@163.com (P.L.); chenjingzhen621@sina.com (J.C.)

**Keywords:** *Gardenia jasminoides*, geniposide, crocin, medicinal ingredients, synthesis pathway, fruit development

## Abstract

*Gardenia jasminoides* fruits are extensively grown worldwide, with a large harvest, and its major medicinal ingredients are geniposide and crocins. Research on their accumulation and biosynthsis-related enzymes is rare. In this study, the accumulation of geniposide and crocin of *G. jasminoides* fruits at different developmental stages were clarified by HPLC. The highest cumulative amount of geniposide was 2.035% during the unripe-fruit period, and the highest content of crocin was 1.098% during the mature-fruit period. Furthermore, transcriptome sequencing was performed. A total of 50 unigenes encoding 4 key enzymes related in geniposide biosynthsis pathways were screened, and 41 unigenes encoding 7 key enzymes in the pathways of crocin were elucidated. It was found that the expression levels of differentially expressed genes of DN67890*_c0_g1_i2*-encoding *GGPS*, which is highly related to geniposide biosynthesis, and DN81253*_c0_g1_i1*-encoding *lcyB*, DN79477*_c0_g1_i2*-encoding *lcyE*, and DN84975*_c1_g7_i11*-encoding *CCD*, which are highly related to crocin biosynthesis, were consistent with the accumulation of geniposide and crocin content, respectively. The qRT-PCR results showed that the trends of relative expression were consistent with transcribed genes. This study provides insights for understanding the geniposide and crocin accumulation and biosynthsis during fruit development in *G. jasminoides*.

## 1. Introduction

**Figure 1:**
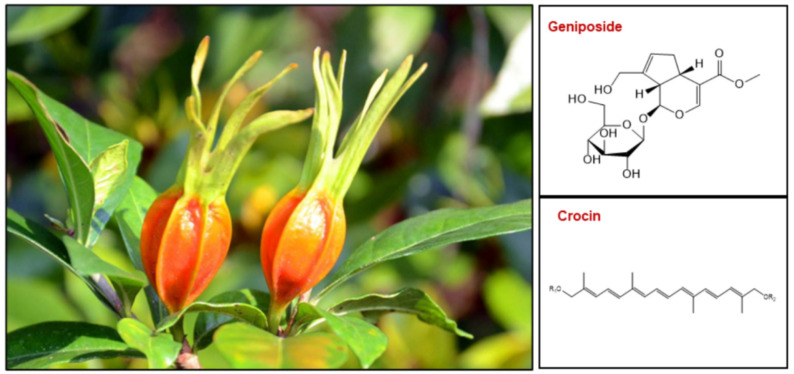
*Gardenia jasminoides* fruit and the chemical structures of geniposide and crocin.

*Gardenia jasminoides* Ellis, an evergreen perennial shrub that belongs to the Rubiaceae family, is a seasonal flowering and fruiting plant [1]. Its white flowers have a heavy fragrance, and are widely used for fresh-cut flowers and garden landscapes [2]. The fruit of *G. jasminoides* contains numerous pharmaceutical functional components and has been widely used as traditional medicine in China and other East Asian countries for a long time [3,4]. The pharmacodynamic chemical components found in *G. jasminoides* fruit mainly include iridoids, monoterpene glycosides, diterpenoids, triterpenoids, organic acid, and flavonoids [5,6,7]. Among them, geniposide and crocin are the two most important bioactive compounds, known for their high medicinal value and significant application potential (Figure 1).

Significant efforts have been made in the past few decades to understand the properties of geniposide and crocin [8,9,10,11]. Geniposide is the main component of iridoid glycosides isolated from *G. jasminoides* fruit, which can reduce the content of alanine transferase and aspartate transferase in serum. Hence, its pharmacological activities, such as anti-diabetic, anti-oxidant, anti-proliferative, and neuro-protective activities, play vital roles in the treatment of various diseases [12,13]. Nowadays, geniposide is expected to become a natural candidate compound to replace antibiotics or hormonal drugs [12]. Furthermore, geniposide also serves as an intermediate for the preparation of natural non-toxic colorant [14,15]. Crocin, another special component in *G. jasminoides* fruit, belongs to the diterpenoid compound family [16,17]. Crocin is the only water-soluble carotenoid, and its natural contents are the highest in both *Crocus sativus* and *G. jasminoides* [18,19]. It has been formally confirmed to have a wide range of pharmacological activities, such as anti-tumor, anti-inflammatory, and anti-oxidant activities, and uses in the treatment of neurodegenerative diseases such as Alzheimer’s disease, depression, sleep loss, learning, memory, and cognitive disorders [20]. It is known that natural products are crucially important resources for new drug research and development with multiple potential health benefits [21]. To date, the demand of *G. jasminoides* fruit has been growing urgently in the marketplace since geniposide and crocin have been widely desired in the fields of medicine, food, and the chemical industry [1,22]. However, the scarcity of geniposide and crocin resources greatly limits their use in new drug development and clinical application.

So far, research on *G. jasminoides* has focused on cultivation, breeding, pigment extraction, and medicinal effects [9,23,24,25,26]. However, the utilization and exploitation of *G. jasminoides* fruit are still limited. In particular, the pharmaceutical quality of herbal-medicine is highly dependent on raw ingredients. Hence, breeding superior quality varieties of *G. jasminoides* should be given more attention [15]. In addition, research on the accumulation of bioactive substances such as geniposide and crocin in *G. jasminoides* fruit during developmental stages has been rarely reported. In recent years, researchers have explored some biosynthsis-related enzymes (*DXS*, *DXS*, *GPPS*, *G10H*, *IS*, *UGTs*, *crtB*, *PDS*, *ZDS*, *lcyB*, *lcyE*, *LUT*, and *CCD*) and their coding genes involved in the iridoid terpenoids pathway and carotenoid pathway in plants such as *Salvia miltiorrhiza*, *Rehmannia glutinosa*, *Arabidopsis thaliana*, *Citrus* sp., *Crocus sativus*, etc. [27,28,29,30]. However, while different types of natural bioactives are produced through a series of enzymatic reactions, the key enzymes in geniposide and crocin biosynthesis are not fully understood, and the regulatory mechanism of geniposide and crocin in *G. jasminoides* fruit has been rarely discussed [31]. The lack of such fundamental research has caused the unreasonable application of *G. jasminoides* resources to a certain extent. Advances in RNA-sequencing technology, combined with multi-omics and molecular biology research methods, have made remarkable progress in the study of biosynthesis of bioactive substances. Breakthroughs have been achieved in the biosynthesis of rare natural products such as artemisinic acid and paclitaxel [32,33]. This greatly promotes the related study on geniposide and crocin biosynthesis.

In this study, the fruits of the Linhai No. 1 cultivar of *G. jasminoides* were selected as the experimental materials. These fruits have undergone testing through the Shenzhou No. 6 spaceflight breeding program in China, which has shown their advantages in terms of excellent quality, large size, high yield, and high pigment content. The objective of this study was to investigate the dynamic changes in geniposide and crocin content during fruit development, and to explore their cumulative patterns and the key enzymes involved in their biosynthesis in *G. jasminoides* fruit development. The aim of this study was to provide an overview for the development and utilization of geniposide and crocin, and to offer further insights for a better understanding and in-depth development of *G. jasminoides* through genetic breeding.

## 2. Results

### 2.1. Morphological Characteristics of G. jasminoides Fruit

**Figure 2:**
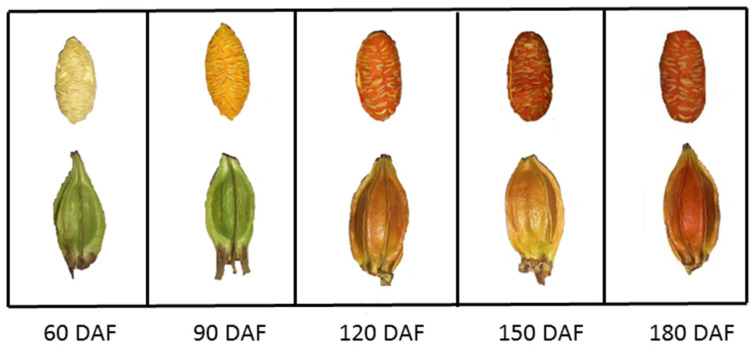
Shifting colors and morphological traits of *G. jasminoides* fruit. Note: The sarcocarps (fruits with peeled-off husks) are in the top row; the intact fruits are in the bottom row. DAF:day after flowering.

The appearance of *G. jasminoides* fruit from five representative developmental stages (60 DAF, 90 DAF, 120 DAF, 150 DAF and 180 DAF) were selected and observed, as shown in Figure 2. The shape of *G. jasminoides* fruit in each period was to be distinctly and long oval. The pericarp of the fruit was bluish green at 60 DAF, then changed to orange-yellow at 120 DAF and orange-red when fully matured at 180 DAF. The sarcocarp was ivory-white at 60 DAF, and the coloration period started at around 90 DAF (about mid-August). At 180 DAF (about mid-November), the sarcocarp and husk were completely colored orange, and the surface of the fruits was slightly shiny with wing-shaped vertical edges, indicating the ripening of the *G. jasminoides* fruit.

**Figure 3:**
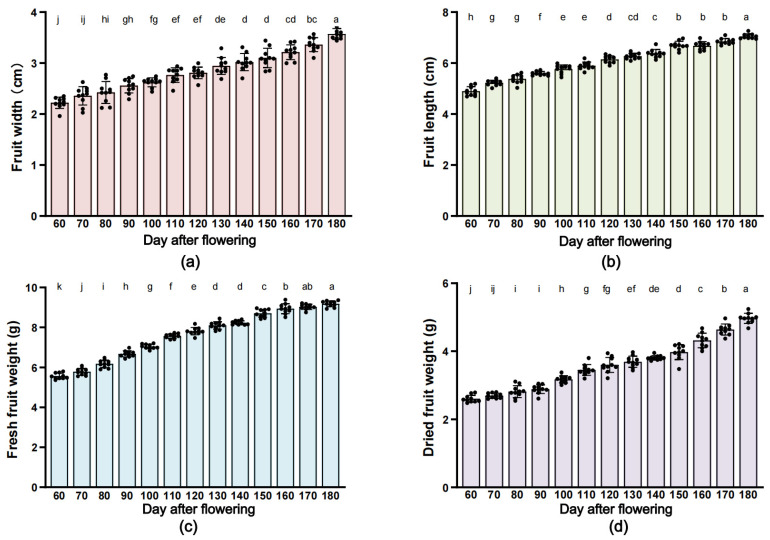
Phenotypic trait of *G. jasminoides* fruit development. Note: Data values are presented from ten biological replicates. One-way analysis was used to test the significant difference, and different lowercase letters in the module indicate significant difference (*p* < 0.05). The morphological changes of fruit width (**a**), fruit length (**b**), fresh fruit weight (**c**) and dried fruit weight (**d**) of *G. jasminoides* fruit during different developmental periods.

During the *G. jasminoides* fruit growth, the phenotypic traits including fruit width, fruit length, fresh fruit weight, and dried fruit weight fruit were determined every 10 days, and the results are shown in Figure 3. The fruit width (Figure 3a) has grown from 2.20 ± 0.11 cm to 3.57 ± 0.11 cm, and the fruit length (Figure 3b) has grown from 4.90 ± 0.18 cm to 7.05 ± 0.10 cm. The fruit weight tended to increase synchronously with fruit length, and both the fresh fruit weight (Figure 3c) and dried fruit weight (Figure 3d) continued to increase slowly until the mature period at 180 DAF, reaching a maximum of 9.18 ± 0.15 g, and 4.97 ± 0.15 g per fruit respectively, and each of them with a net gain of 3.61 g and 2.36 g over the whole fruit development period, respectively.

### 2.2. Dynamic Accumulation of Geniposide and Crocin during G. jasminoides Fruit Growth

**Figure 4:**
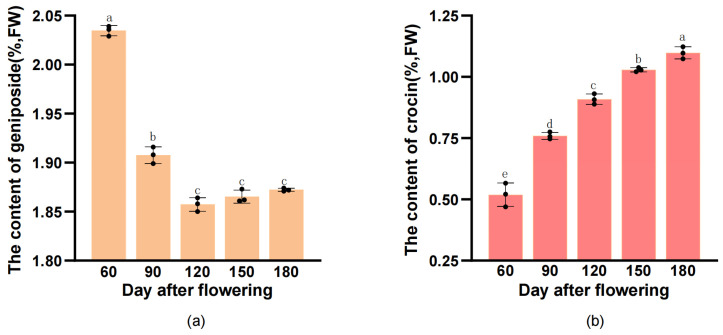
Dynamic accumulation of geniposide and crocin content. Note: Data values are presented from three biological replicates. One-way analysis was used to test the significant difference, and different lowercase letters in the module indicate significant differences (*p* < 0.05). The content changes of geniposide (**a**) and crocin-Ⅰ (**b**) in *G. jasminoides* fruit during different developmental periods.

The geniposide content in *G. jasminoides* fruit was determined by HPLC (Figure 4a); the cumulative amount of geniposide showed a trend of decrease from 60 DAF to 120 DAF, then kept steady during the fruit ripening stages (from 120 to 180 DAF). The highest concentration was 2.035 ± 0.004% in the early stage at 60 DAF when the fruit was young and bluish green. The crocin-I content in *G. jasminoides* fruit among the five developmental periods is shown as Figure 4b. A continuous increase from 0.519 ± 0.039% in young fruit (60 DAF) to the highest cumulative amount of 1.098 ± 0.020% in matured fruit (180 DAF) was observed.

### 2.3. Analysis of DEGs in Geniposide and Crocin Biosysnthsis

To further explore the molecular regulation mechanism in geniposide and crocin biosysnthsis, and to excavate the related gene-encoding key enzymes in *G. jasminoides* fruit, transcriptome sequencing was performed on the three representative developmental stages (T1:60 DAF, T2:120 DAF, and T3:180 DAF) of *G. jasminoides* fruit using the RNA-Seq technique.

#### 2.3.1. Sequencing, Assembly, Function Annotation, Classification and DEGs Analysis

In this study, a total of 62,886,875 clean reads from 64,341,066 raw reads with an average 93.99% of Q30 and 44.46% of GC content were obtained. After filtration and culling, about 298,869 unigenes with an average length of 510 bp, N50 length of 567 bp and N90 length of 246 bp were obtained by transcriptome assembly (Table S2). The clean reads were mapped to the assembled unigenes, with an average alignment rate of 77.75%, and the number of unigenes decreased continuously, indicating that the assembly result could be used for further analysis (Figure S1).

**Figure 5:**
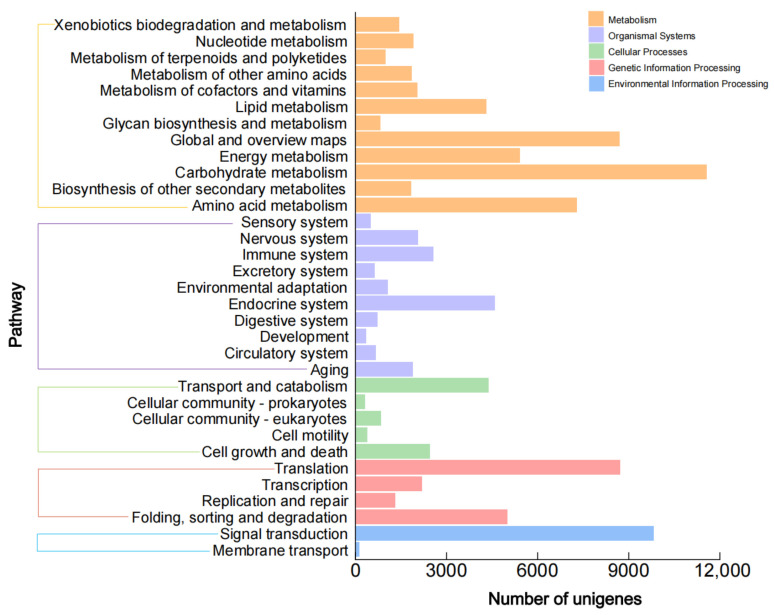
Unigenes classifid by KEGG pathways of *G. jasminoides* fruit.

Blast was performed for the unigenes’ functional annotation. The functional annotations of unigenes were obtained by comparing public databases (NR, Swiss prot, Pfam, GO, and KEGG). Among them, unigenes annotated to the NR database accounted for 51.07% (Table S3). Comparing these unigenes in the GO protein database, the unigenes of functional annotation were divided into three main categories (Figure S2), and the biological processes had the largest quantity with 13 items. Notably, unigenes related to metabolic process accounted for 50.26%. Furthermore, the metabolic pathways of unigenes were compared in the KEGG protein database (Figure 5). A total of 46,940 unigenes were successfully annotated into 284 metabolic pathways in 32 subclasses of 5 categories.

**Figure 6:**
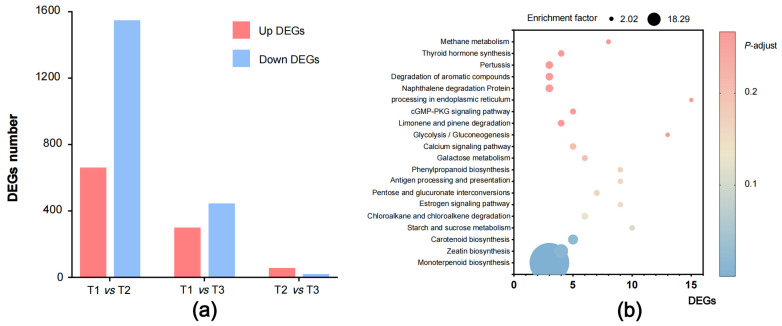
DEGs analysis of *G. jasminoides* fruit. Note: DEGs analysis of *G. jasminoides* fruit at three different developmental periods (**a**) and DEGs annotated by KEGG enrichment analysis in ‘T1 vs. T3’ (**b**).

The comparative transcriptome analysis of *G. jasminoides* fruit at three representative development periods was performed to find out the DEGs related to geniposide and crocin biosynthesis. A total of 2209, 745, and 74 DEGs were found in the ‘T1 vs. T2’, ‘T1 vs. T3’ and ‘T2 vs. T3’ groups, respectively (Figure 6a). There were 661 unigenes up-regulated and 1548 unigenes down-regulated in ‘T1 vs. T2’, and 299 unigenes up-regulated and 446 unigenes down-regulated in ‘T1 vs. T3’, indicating that more DEGs were down-regulated than up-regulated in these two groups. The DEGs in ‘T2 vs. T3’ tended to be much fewer, with only 55 unigenes up-regulated and 19 unigenes down-regulated. The KEGG enrichment analysis was subsequently conducted on the DEGs obtained in ‘T1 vs. T3’ group (Figure 6b) and ‘T1 vs. T2’ group (Figure S3). The top 20 pathways were listed, of which the unigene numbers of monoterpenoid biosynthesis, zeatin biosynthesis, and carotenoid biosynthesis pathways were the largest categories in the ‘T1 vs. T3’ group (corrected *p*-value < 0.05), and there were three DEGs related to terpenoid biosynthesis in upstream pathways with an enrichment factor of 166. In the ‘T1 vs. T2’ group, eight DEGs related to the carotenoid pathway with an enrichment factor of 6.18 were found, which indicated that there were strong activities in the anabolism of geniposide and crocin during *G. jasminoides* fruit ripening.

#### 2.3.2. Candidate Genes Involved in Geniposide and Crocin Biosynthesis in *G. jasminoides* Fruit

**Figure 7:**
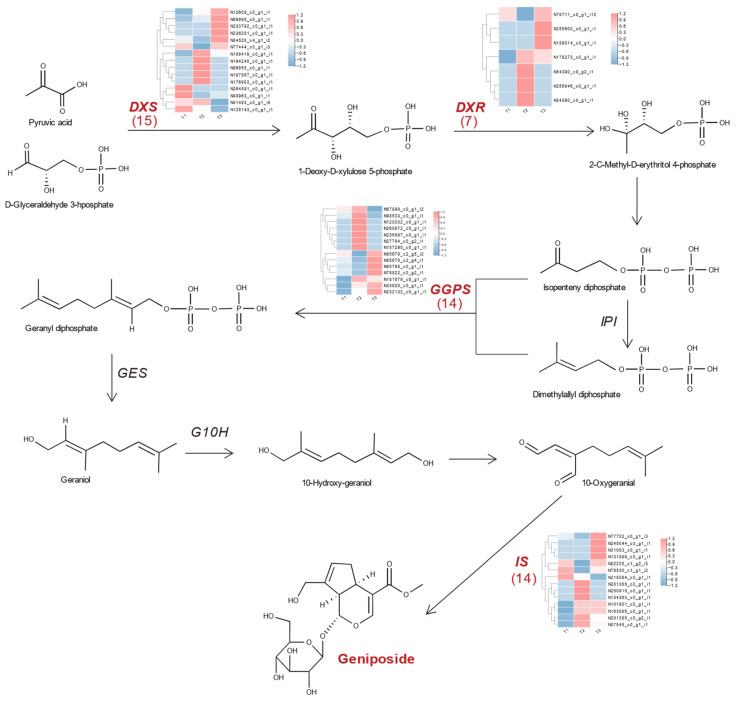
The schematic diagram of the pathway of geniposide biosynthesis in *G. jasminoides* fruit.

Geniposides are iridoid glycosides which are synthesized through the terpene metabolic pathway in upstream, and these enzymes (*DXS, DXR, GPPS, IS*, etc.) play crucial roles involved in geniposide biosynthesis. Therefore, based on the KEGG function annotation, the genes encoding these four key enzymes involved in biosynthesis of geniposide were excavated (Table 1). There are 15 unigenes coding 1-deoxy-D-xylulose-5-phosphate synthase (*DXS*), which is a crucial enzyme at the beginning of catalyzing of terpenoid biosynthesis. There are seven unigenes coding 1-deoxy-D-xylulose-5-phosphate reductoisomerase (*DXR*). In addition, 14 unigenes of dimethylallyltranstransferase (*GPPS*) and 14 unigenes of 8-oxocitronellyl enol synthase (*IS*) are found involved in the biosynthesis of geniposide. Furthermore, the pathway involved in geniposide biosynthesis of *G. jasminoides* fruit was demonstrated by displaying these enzymes and the FPKM of their DEGs identified in our study (Figure 7).

**Figure 8:**
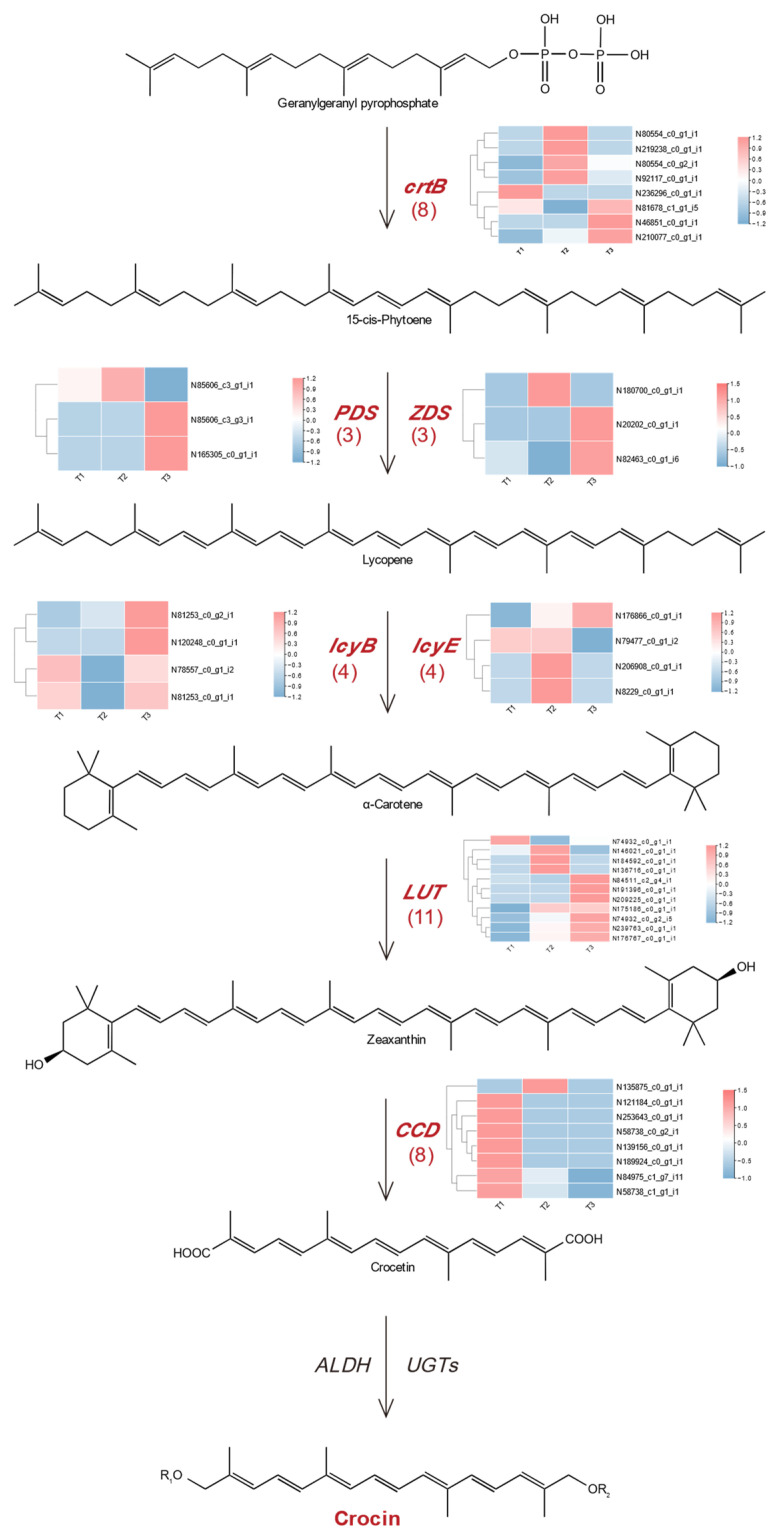
The schematic diagram of the pathway of crocin biosynthesis in *G. jasminoides* fruit.

Crocin belongs to the carotenoid compounds, and the pathway of carotenoids was deduced by previous studies through isopentadienoid compound synthesis. In this study, the pathway of crocin synthesis involved in *G. jasminoides* fruit growth was inferred by carotenoid biosynthesis. The *crtB*, *PDS*, *ZDS*, *lcyB*, *lcyE*, *LUT*, and *CCD* were distinguished as key enzymes that were relatively abundant; several unigenes underlying these enzymes related to crocin biosynthesis of *G. jasminoides* fruit were found (Table 1). There were eight unigenes coding in 15-cis-phytoene synthase (*crtB*). It was the catalyst for the production of 15-cis-phytoene, which is closely related to pigment production and promotes the synthesis of corresponding carotenoids. There were three unigenes coding in phytoene desaturase (*PDS*), while *PDS* contributed to enzyme-catalyzed reaction in the process of binding to the plasma membrane through transfer into the cytoplasm. Three unigenes were identified to encode 9,9′-dicis-ζ-carotene desaturase (*ZDS*); the *ZDS* and *PDS* are vital enzymes for desaturating lycopene production. There are four unigenes to encode lycopene β-cyclase (*lcyB*), four unigenes to encode lycopene ε-cyclase (*lcyE*), 11 unigenes to encode carotenoid ε-hydroxylase (*LUT*), and eight unigenes to encode crocetin dialdehyde synthase (*CCD*), respectively. Therefore, we constructed the crocin biosynthetic pathway of *G. jasminoides* fruit by displaying the key enzymes, gene names and their FPKM values in Figure 8.

#### 2.3.3. Analysis of Key Genes Involved in Geniposide and Crocin Biosynthesis

The previous analysis in this study showed that the geniposide and crocin content of *G. jasminoides* fruit was significantly different between T1 and T3; meanwhile, there were vast amounts of DEGs annotated in the ‘T1 vs. T2’ group and the ‘T1 vs. T3’ group. Therefore, the key genes were excavated and their FPKM and fold change values were counted (Table 2).

The FPKM values of genes related to geniposides biosynthesis shown in ‘T1 vs. T3’, DN80963*_c0_g1_i1* (*DXS*) was down-regulated by 3.0 times, DN67890*_c0_g1_i2* (*GGPS*) down-regulated by 9.3 times, and DN78530*_c1_g1_i2* (*IS*) down-regulated by 1.3 times. We also found the fold change of these unigenes had the larger multiple change in the ‘T1 vs. T3’ group. That might suggest a rapid decrease in geniposide content during fruit development. It was noteworthy that the gene of DN79711*_c0_g1_i12* (*DXR*) showed a trend of up-regulation by 1.5 times in ‘T1 vs. T3’, and down-regulation by 5.8 times in ‘T1 vs. T2’. These results are basically in agreement with the trend of geniposide content at the growth period of *G. jasminoides* fruit. Therefore, we believe that these unigenes, especially DN67890*_c0_g1_i2* (*GGPS*), played key roles in the biosynthesis of geniposide.

In addition, the FPKM values of key genes in the crocin pathway were calculated (Table 2). The results showed that DN81678*_c1_g1_i5* (*crtB*) was significantly up-regulated during whole developmental stages, and the expression level was increased 1.3 times from the 60 DAF to 180 DAF, and 2.1 times from the 120 DAF to 180 DAF, which was consistent with the change trend of the crocin content. In ‘T1 vs. T3’, DN85606*_c3_g3_i1* (*PDS*), DN82463*_c0_g1_i6* (*ZDS*), DN81253*_c0_g1_i1* (*lcyB*), DN79477*_c0_g1_i2* (*lcyE*), DN84511*_c2_g4_i1* (*LUT*), and DN84975_c1_g7_i11 (*CCD*) were up-regulated, by 2.3 times, 1.9 times, 1.1 times, 1.2 times, 3.3 times and 1.1 times, respectively; this closely followed the trend of continuous increase in crocin content during the ripening of *G. jasminoides* fruit. Notably, the large fold changes of DN81253*_c0_g1_i1* (*lcyB*), DN79477*_c0_g1_i2* (*lcyE*) and DN84975*_c1_g7_i11* (*CCD*) were found in ‘T1 vs. T3’, indicated that *lcyB*, *lcyE* and *CCD* may be the key genes related to crocin biosynthesis.

### 2.4. Validation of RNA-Seq Data by qPCR

**Figure 9:**
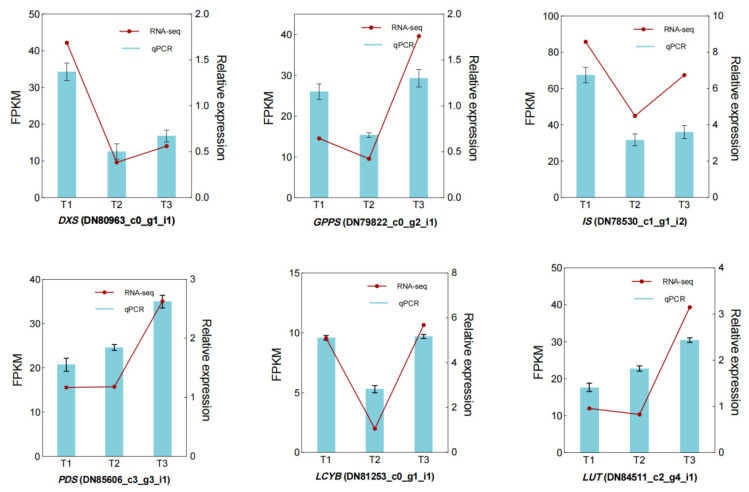
Relative expression levels of DEGs in three development periods of *G. jasminoides* fruit.

Six unigenes coding key enzymes of geniposide and crocin biosynthesis were randomly selected for qPCR to verify the accuracy of the RNA-seq result. The relative expression levels of these genes were different in different *G. jasminoides* fruit developmental periods, and the expression patterns were consistent with the data of RNA-seq (Figure 9). Therefore, our RNA-seq results provided reliable data for future research on key enzymes of geniposide and crocin biosynthesis in *G. jasminoides* fruit.

## 3. Discussion

### 3.1. Phenotypic Results

*G. jasminoides* fruit has been applied as a dietary supplement and traditional Chinese medicine for thousands of years. In this study, the *G. jasminoides* fruits from different developmental stages were collected from 60 DAF to 180 DAF, which are associated with fruitlets in a green pericarp and immature sarcocarp in mid-July to fully matured inner and outer orange-red in mid-November, respectively. The fruit length is between 4.90 ± 0.18 cm and 7.05 ± 0.10 cm, while other cultivars the length is usually 1.4 to 7.0 cm [15,34], which revealed that the *G. jasminoides* fruit of cultivar of Linhai No.1 is larger than other regular cultivars. Accompanied by fruit enlargement and ripening, the fruit increase in weight during the developmental period (120 days); the *G. jasminoides* average weight for a single fruit ranged from 5.57 ± 0.15 g to 9.18 ± 0.15 g (FW), and 2.61 ± 0.10 g to 4.97 ± 0.15 g (DW) respectively. That means the fruit weight showed an absolutely positive correlation with the ripening of the fruit, which was same as in previous studies [35].

### 3.2. Dynamic Accumulation of Geniposide and Crocin

The plant’s metabolites are inseparable from its role as an edible food providing numerous nutrients or as a medical plant exerting special functions. The *G. jasminoides* fruit have a long harvesting time, usually be collected when the fruits turn yellow, around September to November in China [15]. Previous research showed that harvest period crucially influenced the plant’s secondary metabolites [21,36]. Considering the dynamic accumulation of geniposide and crocin in different growth periods of *G. jasminoides* fruit, so it is necessary to explore the accumulation changes of geniposide and crocin in order to find out the optimum harvesting time and to provide supports for the utilization of *G. jasminoides* fruit. In this study, there was a significant difference between geniposide and crocin accumulation of *G. jasminoides* fruit during harvesting periods. According to the HPLC determination, the content of geniposide was the highest (2.035 ± 0.004%) in the fruitlet period. As the fruit developed, it gradually decreased and reached stability during the ripening time. The result is consistent with other researches, which showed that content of geniposide is highest in the green fruit stage [37,38,39]. However, in this study, the content of crocin gradually increased during fruit ripening, and reached its highest (1.098 ± 0.020%) at 180 DAF, which was consistent with the former report of crocin content in *G. jasminoides* revealing a significant increase during fruit ripening [2,16]. That means that the *G. jasminoides* fruit is best harvested in the young fruit period if geniposides are used as the quality evaluation standard, whereas, for the preparation of crocin, its best harvesting time is the fully matured period at 180 DAF (about mid-November).

### 3.3. DEGs Related to Geniposide and Crocin Biosysnthsis

Geniposide is an active pharmaceutical ingredient (API) for the treatment of cardiovascular, cerebrovascular, and hepatobiliary disease, diabetes and other diseases, and crocin is the main medicinal component of *Crocus sativus* and *G. jasminoides*, which has the function of regulating the nerve center and improving memory and cognition [8]. The synthetic pathways of geniposide and crocin have widely attracted researchers’ attention [40]. With the publication of genome and transcriptome sequences of several related species, more and more genes encoding various vital enzymes have been identified [8,31]. In this study, we deciphered the pathway of geniposide and crocin biosysnthesis of *G. jasminoides* fruit by transcriptome analysis. Transcriptome sequencing was performed at three fruit developmental stages (60 DAF, 120 DAF and 180 DAF), and bioinformatic methods such as sequencing evaluation and protein annotation were used to analyze the biosynthesis of geniposide and crocin. GO and KEGG enrichment analysis of DEGs based on time series analysis showed that, the growth of *G. jasminoides* fruit was actually required for the catalysis and regulation of a variety of enzymes, and also involved in a number of secondary metabolite biosynthesis pathways, mainly including terpenoid skeleton biosynthesis, carotenoid biosynthesis, etc. Previous studies showed that *DXS*, *DXR*, *GPPS*, and *IS* had been verified as key enzymes upstream of the terpene biosynthesis, and *crtB*, *PDS*, *ZDS*, *lcyB*, *lcyE*, *LUT*, and *CCD* had been verified as key enzymes of the crocin biosynthesis [10,40,41,42]. Therefore, in this study, four putative enzymes including *DXS*, *DXR*, *GPPS*, and *IS*, related to geniposide biosynthesis pathways and the encoding genes were screened and seven putative enzymes including *crtB*, *PDS*, *ZDS*, *lcyB*, *lcyE, LUT, and CCD* in the pathways of crocin and the encoding genes were elucidated.

This study found that there was a consistent trend of several unigenes encoding enzymes in *DXS*, *GPPS*, and *IS* expression with the content changes in geniposide, mainly including DN80963*_c0_g1_i1* (*DXS*), DN67890*_c0_g1_i2* (*GGPS*), and DN78530*_c1_g1_i2* (*IS*). Their high expression in immature fruit suggested that they are the primary enzymes involved in geniposide biosynthesis. Terpenes are synthesized mainly based on the mevalonate (MVA) pathway and methylerythritol 4-phosphate (MEP) pathway; the reported pathways for terpenoid synthesis showed that *DXS* and *DXR* are the two most critical rate-limiting enzymes in the terpenoid backbone biosynthesis in the MEP pathway in many species [43,44]. Five genes of *DXS* were found in *Salvia miltiorrhiza*, according to their specificity in tissue expression; it is speculated that two genes encoding *DXS* may be involved in terpenoid secondary metabolism [29]. During tomato ripening, the expression level of these genes coding *DXS* was highly correlated with carotenoid accumulation [45]. A study revealed that overexpression of the *DXR* gene in Mentha haplocalyx transgenic plants increased the contents of chlorophyll, carotene and peppermint essential oil [46]. In plants, *GPPS* catalyzes the condensation of precursors to produce GPP, which is a precursor of monoterpenoids. The accumulation of *GPPS* and terpenoids in Rehmannia had a significant correlation [47], which is similar to the result in our study. We also found DN67890*_c0_g1_i2* encoding *GGPS*, extremely significantly down-regulated in ‘T1 vs. T3’, revealed it may be highly related to the biosynthesis of geniposide. The enzyme of *IS* reduces 8-oxogeranial and generates iridoids that are involved in the biosynthesis of many indole alkaloids. In this study, 14 genes encoding *IS* were identified, and a gene (DN78530*_c1_g1_i2*) was significantly expressed, while another study showed 11 candidate genes encoding *IS* were found involved in the iridoids biosynthesis pathway in *G. jasminoides* [8].

Carotenoids are the most widely distributed class pigments in nature, and their synthesis is regulated by a number of metabolic enzyme genes. Crocin compounds are the products of crocin biosynthesis and are often mediated by the carotenoid pathway in *Saffron crocus*, *Crocus sativus*, *Buddleja officinalis* and other plants [8,41,48]. The key gene-encoding enzymes for crocin synthesis in the upstream pathway, including DN81678*_c1_g1_i5* (*crtB*), DN85606*_c3_g3_i1 (PDS*), and DN82463*_c0_g1_i6* (*ZDS*), and key genes encoding enzymes for crocin synthesis in the middle-downstream pathway including DN81253*_c0_g1_i1* (*lcyB*), DN79477*_c0_g1_i2* (*lcyE*), DN84511*_c2_g4_i1* (*LUT*), and DN84975*_c1_g7_i11* (*CCD*) were identified, which showed these genes significantly expressed during the *G. jasminoides* fruit developmental stages. According to the analysis of FPKM values and fold change, we found the DN81253*_c0_g1_i1*-encoding *lcyB*, DN79477_*c0_g1_i2*-encoding *lcyE*, and DN84975*_c1_g7_i11*-encoding *CCD* closely related to the crocin accumulation which was highly expressed in matured fruits. The *lcyB* gene in citrus plays a key role in carotene formation by directing the flow of carotene metabolism to the β-branch, leading to the accumulation of lutein during citrus fruit ripening [28]. Studyof the expression of *lcyB* in watermelon [49] revealed that *lcyB* was significantly expressed in red and pink watermelons, but almost not detected in yellow and white watermelons, which proved that the level of *lcyB* expression regulated the color quality of fruits. The relative expression of *lcyE* genes in different developmental stages of yellow zucchini was significantly higher than that in white zucchini [50]. The expression levels of seven key genes of lycopene metabolism were studied in different developmental stages of tomato fruits. The results showed that *lcyB* genes and *lcyE* genes were involved in lycopene accumulation and regulation of fruit color [51]. Rate-limiting enzymes of *CCD* involved in the biosynthesis of crocin compounds are key enzymes that direct zeaxanthin, an intermediate product of the carotenoid synthesis pathway to crocin synthesis. The gene encoding *CCD* was highly expressed in the orange stage of stigma development in *Crocus sativus*, and the accumulation of crocetin reached the maximum [52]. Our study speculated that the regulation the expression of *lcyB*, *lcyE*, and *CCD* would change the crocin production of *G. jasminoides* fruit.

## 4. Materials and Methods

### 4.1. Plant Materials

The cultivar (Linhai No.1) of *G. jasminoides* was planted at Haitai Bonong Planting Demonstration Base in Yueyang city, China (113°08′ E, 28°80′ N). And about 100 healthy fruits were randomly selected and harvested from 60 DAF to 180 DAF, then properly treated and brought back to the laboratory for further analysis. According to analyze the morphological characteristics on developmental *G. jasminoides* fruit, the five key periods (60 DAF, 90 DAF, 120 DAF, 150 DAF, 180 DAF) were selected to determine bioactive substances, and three key periods (60 DAF, 120 DAF, 180 DAF) were selected to perform integrated transcriptome analysis.

### 4.2. Investigation of Morphological Traits

The morphological traits of *G. jasminoides* fruit were analyzed in the Central Process Lab of Hunan Academy of Forestry. The fruit length (FL) and fruit width (FW) were measured from each period by a Vernier Caliper (precision 0.01 cm), with 10 replicates. The fresh fruit weight (FFW) and dried fruit weight (DFW) were determined using an analytical balance (precision 0.0001 g), with 10 replicates.

### 4.3. Determination of Geniposide and Crocin-Ⅰ Content

The freezedried *G. jasminoides* fruit were ground into powder by a pulverizer and sieved through 24 meshes. The powder was put into an Erlenmeyer flask, added to 10 mL 75% methanol solution (*v*/*v*) to soak within ultrasonic extraction (45 kw∙h^−1^) for 60 min, then centrifuged (4 °C, 8000 r∙min^−1^) for 10 min, and supernatant fluid was taken over 0.22 μm microporous filter membrane for HPLC analysis. Geniposide and crocin-I standards (purity ≥98%; purchased from Shanghai yuanye Bio-Technology Co., Ltd., Shanghai, China) were weighed accurately, and put into 10 mL volumetric bottle, dissolved with 75% methanol to volume. The determination was performed on an LC-20AT HPLC analyzer (Agilent Technologies Co., Ltd., Santa Clara, CA, USA) with an Agilent ZORBAX 300SB-C18 column (250 mm × 4.6 mm, 5 μm); the column temperature was 25 °C; The flow rate was 1 mL∙min^−1^; the injection volume was 10 uL; the mobile phase (A:B = 15:85, *v*/*v*) was acetonitrile (A) with 0.1% phosphoric acid (B); and the detection wavelength of geniposide was 238 nm. The mobile phase (A:B = 25:75, *v*/*v*) was acetonitrile (A) with 0.1% phosphoric acid (B), and the detection wavelength of crocinwas 440 nm. Each test was conducted with three biological replicates.

### 4.4. RNA Extraction and Sequencing

Total RNA of three periods (60 DAF, 120 DAF, 180 DAF) of *G. jasminoides* fruit was extracted with a Plant Total RNA Isolation Kit (Number:B518631, Sangon Biotech., Shanghai, China), and the purity and concentration of total RNA were detected with a Nanodrop 2000 ultramicro spectrophotometer. The cDNA library was prepared according to the mRNA-Seq sample preparation kit (Illumina Biotechnology Company, San Diego, CA, USA). Illmina2500 (HiSeq 2500, Illumina) sequencing platform was used to sequence the constructed cDNA library. We removed the adapter from the raw reads and filtered out the low-quality reads to get clean reads. We reassembled all the clean read fragments by using Trinity software. Overlapping regions with certain-length reads were obtained and assembled into fragment transcripts. We eliminated the short genes of each transcript, and kept the longest transcripts to compile them into non-redundant unigenes by alignment.

### 4.5. Function Annotation and Analysis of Differentially Expressed Genes

The functional annotation was obtained from comparing the assembled RNA base sequences with protein databases using Blast X (NCBI Blast 2.2.29+). The transcriptome sequencing data in this study were compared with public protein databases including the Non-Redundant Protein Sequence Database (NR), SWISS-PROT, protein families (Pfam), Gene Ontology (GO) and Kyoto Encyclopedia of Genes and Genomes (KEGG). Expectation-Maximization (RSEM) was used to compare the RNA-Seq data after quality control with the transcriptome assembly sequences to estimate the unigene expression levels of FPKM (Fragments per kilobase million). The differentially expressed genes (DEGs) between different development periods were screened using DESeq2 based on negative binomial distribution with certain screening criteria (*p*-adjust < 0.05, |log2 Fold Change| ≥ 1). The *p*-value obtained from the statistical test was corrected by BH (FDR correction with Benjamini/Hochberg) to obtain *p*-adjust.

### 4.6. Validation of RNA-Seq by Quantitative PCR

Six key genes related to biosynthesis of geniposide (DN80963*_c0_g1_i1*, DN79822*_c0_g2_i1*, DN7853*0_c1_g1_i2*) and crocin (DN85606*_c3_g3_i1*, DN81253*_c0_g1_i1*, DN8451*1_c2_g4_i1*) were selected as target genes (Table S1), and primers were designed using Primer Premier 5.0. ACTR 2 (actin-related protein 3) was used as the internal reference gene. The reaction mixtures were prepared as described in Table 3. Quantitative PCR was performed to investigate the expression levels of the target genes, and each reaction was repeated three times. Quantitative real-time PCR was performed using the following protocol: 10 s pre-denaturation at 95 °C; 40 cycles of 95 °C for 10 s, and 60 °C for 30 s. Upon completion of the cycle, the melting curve was amplified using the following procedure: 95 °C for 15 s; 60 °C for 60 s; and 95 °C for 15 s.

### 4.7. Statistical Analysis

All data analysis was performed using IBM SPSS Statistics 22.0 software and the data presented as mean ± SD (standard deviation). The statistical differences between the groups were tested by one-way analysis of variance (ANOVA). The chemical structures were drawn by ChemDraw 22.0 software.

## 5. Conclusions

In summary, the content dynamic changes and gene expression of geniposide and crocin in *G. jasminoides* fruit were analyzed, and the biosynthetic pathways of geniposide and crocin were systematically clarified. With the fruit ripening, there were differently dynamic changes in the accumulation of geniposide and crocin-I, which showed opposite accumulation patterns. The geniposide content was at its maximum of 2.035% in the green fruit period at 60 DAF, while the crocin content was at its the maximum of 1.098% in the fully matured period at 180 DAF. Based on the analysis of morphological characteristics and accumulation of *G. jasminoides* fruit, the optimal harvesting time were determined for different purposes. The DEGs of *G. jasminoides* fruit were identified by the RNA-Seq technique among different developmental periods. Between 60 vs. 120 DAF, 60 vs. 180 DAF, and 120 vs. 180 DAF there were 661, 229 and 55 unigenes upregulated, and 1548, 446 and 19 differential genes down-regulated, respectively. A total of 50 unigenes encoding four enzymes, including *DXS*, *DXR*, *GPPS*, and *IS* related to the geniposide biosynthetic pathway were screened, and 41 unigenes encoding seven enzymes including *crtB*, *PDS*, *ZDS*, *lcyB*, *lcyE*, *LUT*, and *CCD* in the carotenoid pathway of crocin were elucidated. We found DN67890*_c0_g1_i2* encoding *GGPS* may be highly related to the biosynthesis of geniposide, and DN81253*_c0_g1_i1* encoding *lcyB*, DN79477*_c0_g1_i2* encoding *lcyE*, and DN84975*_c1_g7_i11* encoding *CCD* may be highly related to crocin biosynthesis. This work will enhance the current understanding of accumulation and associated biosynthesis mechanisms of geniposide and crocin in *G. jasminoides* fruit.

## Figures and Tables

**Table 1:** Genes encoding key enzymes related to biosynthesis of geniposide and crocin.

Category	Name	Symbol	Enzyme No.	Unigene Number
Geniposide	1-deoxy-D-xylulose-5-phosphate synthase	*DXS*	EC:2.2.1.7	15
	1-deoxy-D-xylulose-5-phosphate reductoisomerase	*DXR*	EC:1.1.1.267	7
	dimethylallyltranstransferase	*GPPS*	EC:2.5.1.1	14
	8-oxocitronellyl enol synthase	*IS*	EC:1.3.1.122	14
Crocin	15-cis-phytoene synthase	*crtB*	EC:2.5.1.32	8
	phytoene desaturase	*PDS*	EC:1.3.99.29	3
	9,9′-dicis-ζ-carotene desaturase	*ZDS*	EC:1.3.5.6	3
	lycopene β-cyclase	*lcyB*	EC:5.5.1.19	4
	lycopene ε-cyclase	*lcyE*	EC:5.5.1.18	4
	carotenoid ε-hydroxylase	*LUT*	EC:1.14.14.158	11
	crocetin dialdehyde synthase	*CCD*	EC:1.13.11.84	8

**Table 2:** The DEGs involved in geniposide and crocin biosynthesis.

Category	Enzymes	Unigenes	FPKM			Fold Change (T1 vs. T3)
			T1	T2	T3	
Geniposide	*DXS*	DN80963*_c0_g1_i1*	42.17	9.62	14.02	2.6618
	*DXR*	DN79711*_c0_g1_i12*	29.47	5.04	44.40	0.5532
	*GPPS*	DN85670*_c2_g5_i2*	90.39	6.66	297.59	0.2600
		DN67890_*c0_g1_i2*	5.19	10.36	0.56	7.4014
		DN79822*_c0_g2_i1*	14.52	9.56	39.63	0.3253
	*IS*	DN78530*_c1_g1_i2*	85.83	44.95	67.42	1.1134
Crocin	*crtB*	DN81678*_c1_g1_i5*	51.93	27.67	65.78	0.6261
	*PDS*	DN85606*_c3_g3_i1*	15.58	15.76	35.09	0.3805
	*ZDS*	DN82463*_c0_g1_i6*	18.09	12.80	34.09	0.4459
	*lcyB*	DN81253*_c0_g1_i1*	9.56	1.99	10.66	0.7729
	*lcyE*	DN79477*_c0_g1_i2*	8.88	8.91	10.06	1.2696
	*LUT*	DN84511*_c2_g4_i1*	11.97	10.36	39.36	0.2633
	*CCD*	DN84975*_c1_g7_i11*	12.64	11.32	13.81	2.7433

Note: FPKM, Fragment Per Kilobase per Million bases; T1, T2 and T3 represent 60 DAF, 120 DAF and 180 DAF, respectively.

**Table 3:** qPCR constituents.

Reaction Reagent	Sample Volume
2× ChamQ SYBR qPCR Master Mix	10 μL
Primer1 (10 μM)	0.4 μL
Primer2 (10 μM)	0.4 μL
50× ROX Reference Dye 1	0.4 μL
Template DNA/cDNA	2.0 μL
Double distilled H_2_O	6.8 μL

## Data Availability

The data that support the findings of this study are available from the corresponding author upon reasonable request.

## Supplementary Materials

The following supporting information can be downloaded at: https://www.mdpi.com/article/10.3390/plants12112209/s1, Figure S1: Frequency of *G. jasminoides* fruit unigenes; Figure S2: Gene Ontology (GO) pathways of *G. jasminoides* fruit unigenes; Figure S3: DEGs annotated by KEGG enrichment analysis in ‘T1 vs. T2’; Table S1: qRT-PCR validation primer sequences; Table S2: Assembling of the sequencing data; Table S3: Functional annotation of *G. jasminoides* fruit in public protein databases; Table S4: The assembled genes.
