# Supplementary material for: Elucidation of Geniposide and Crocin Accumulation and Their Biosysnthsis-Related Key Enzymes during Gardenia jasminoides Fruit Growth
Source: Plants (Basel). 2023 Jun 3;12(11):2209. doi: 10.3390/plants12112209 (PMC10255275; doi:10.3390/plants12112209)
Supplement: Supplementary file 1 [file plants-12-02209-s001.zip › Figure S1-S3 Table S1-S3.pdf]

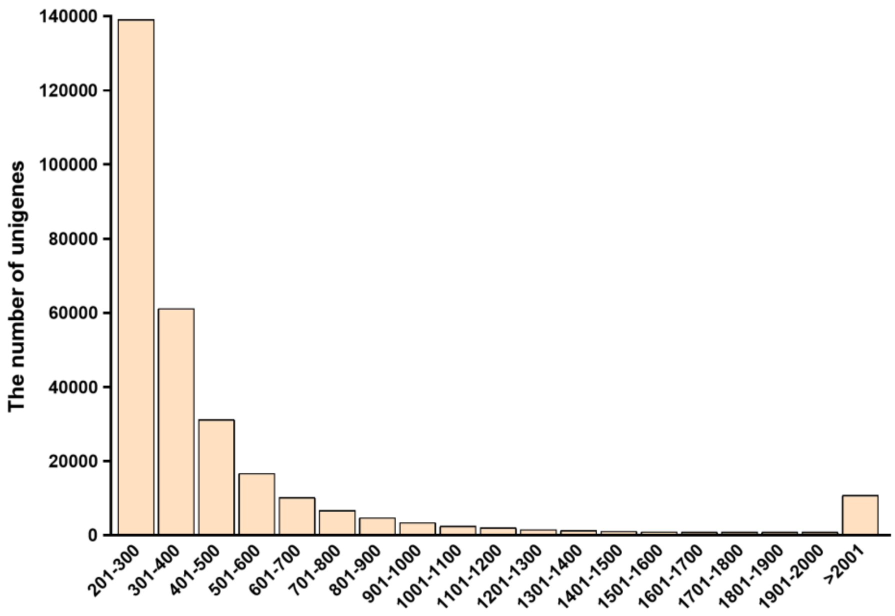

Figure S1. Frequency of *G. jasminoides* fruit unigenes

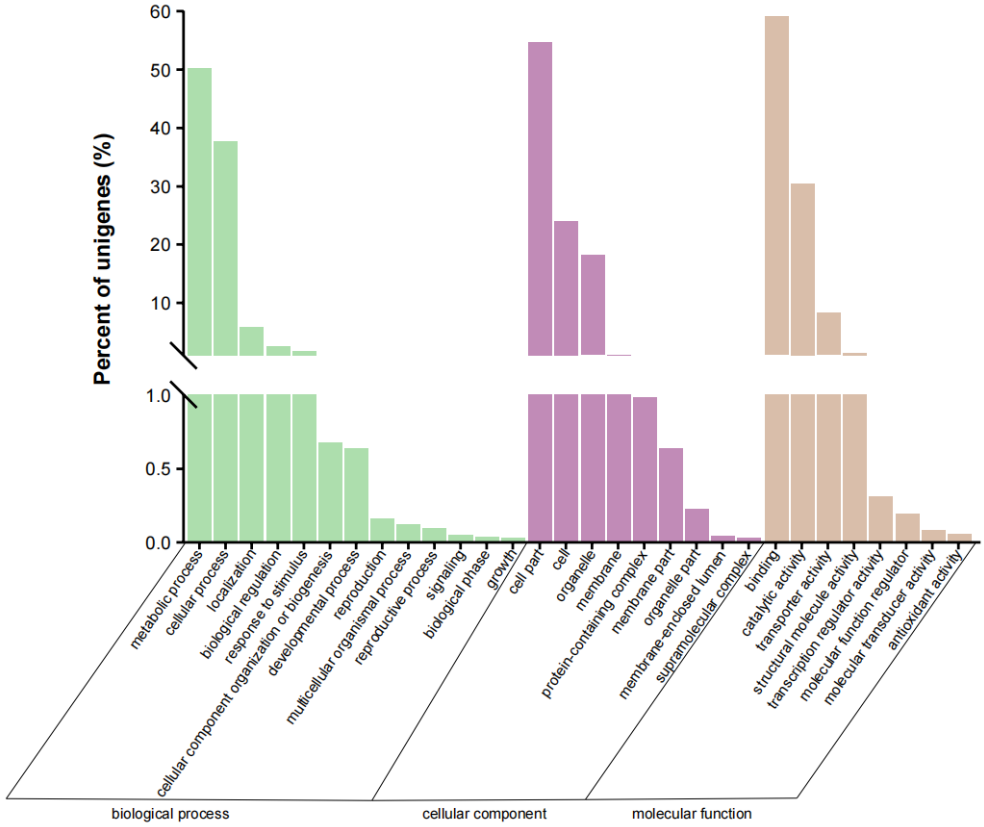

Figure S2. Gene Ontology (GO) pathways of *G. jasminoides* fruit unigenes

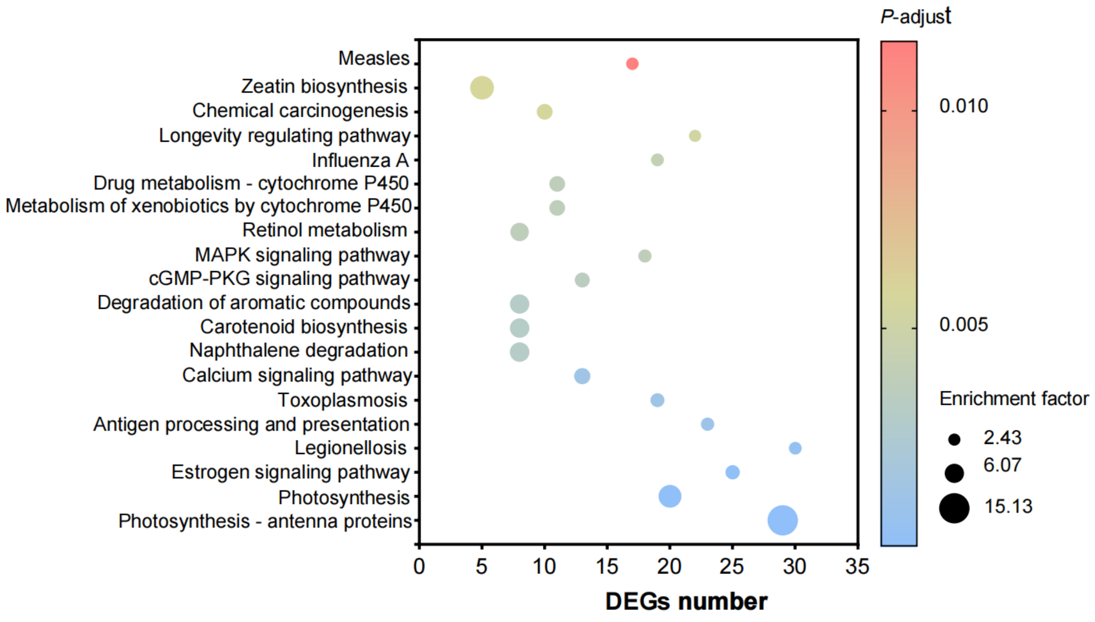

Figure S3. DEGs annotated by KEGG enrichment analysis in‘ T1 vs T2 ’

Table S1 qRT-PCR validation primer sequences

Gene ID	Forward (5'-3')	Reverse (5'-3')
DN80963_c0_g1_i1	CATGGTGGTGATGGCTCCTT	CCCTCTCGGATAACGGAAGC
DN79822_c0_g2_i1	GTGGATGGCCAATTCAGGGA	TATAGCTCCACACGCAGCAG
DN78530_c1_g1_i2	GATGGGGATGTGTTGCTCCA	ACGGAGCGCCTTTCCATTTA
DN85606_c3_g3_i1	TCGATGGAAGGTGCTGTTCT	GCTTGCCTCAGGTATCCTCT
DN81253_c0_g1_i1	TGGCATGGAGACCTTACTGAA	GCATGGCAAGCTCTCCTAGT
DN84511_c2_g4_i1	GTCACTGCTCTGCTGCTACA	GAAGCAGAGGAAGACTCGCA

Table S2. Assembling of the sequencing data

Statistics	Number	Total length (bp)	Average length (bp)	N50 (bp)	N90 (bp)
Transcripts	384 421	321 711 569	836	1859	288
Unigenes	298 869	152 447 815	510	567	246

Table S3. Functional annotation of *G.jasminoides* fruit in public protein databases

Annotated Databases	Total of Unigenes	Number of Unigenes	Percentage (%)
Annotated in NR	298 869	152 647	51.07%
Annotated in Swiss_prot	298 869	92 529	30.96%
Annotated in pfam	298 869	91 287	30.54%
Annotated in GO	298 869	23 358	7.82%
Annotated in KEGG	298 869	46 940	15.71%
